# Novel *EDA* or *EDAR* Mutations Identified in Patients with X-Linked Hypohidrotic Ectodermal Dysplasia or Non-Syndromic Tooth Agenesis

**DOI:** 10.3390/genes8100259

**Published:** 2017-10-05

**Authors:** Binghui Zeng, Qi Zhao, Sijie Li, Hui Lu, Jiaxuan Lu, Lan Ma, Wei Zhao, Dongsheng Yu

**Affiliations:** 1 Guanghua School of Stomatology, Hospital of Stomatology, Guangdong Provincial Key Laboratory of Stomatology, Sun Yat-sen University, Guangzhou 510055, China; zengbh@mail3.sysu.edu.cn (B.Z.); lisijie@mail2.sysu.edu.cn (S.L.); luhui7@mail2.sysu.edu.cn (H.L.); lujxuan@mail.sysu.edu.cn (J.L.); malan6@mail.sysu.edu.cn (L.M.); 2 Department of Oncology, Xianning Central Hospital, The First Affiliated Hospital of Hubei University of Science and Technology, Xianning 437100, China; zhaoqihb@hotmail.com

**Keywords:** tooth agenesis, hypodontia, ectodermal dysplasia, *EDA*, *WNT10A*, *EDAR*, exome sequencing

## Abstract

Both X-linked hypohidrotic ectodermal dysplasia (XLHED) and non-syndromic tooth agenesis (NSTA) result in symptoms of congenital tooth loss. This study investigated genetic causes in two families with XLHED and four families with NSTA. We screened for mutations of *WNT10A*, *EDA*, *EDAR*, *EDARADD*, *PAX9*, *MSX1*, *AXIN2*, *LRP6*, and *WNT10B* through Sanger sequencing. Whole exome sequencing was performed for the proband of NSTA Family 4. Novel mutation c.1051G>T (p.Val351Phe) and the known mutation c.467G>A (p.Arg156His) of *Ectodysplasin A* (*EDA*) were identified in families with XLHED. Novel *EDA receptor* (*EDAR*) mutation c.73C>T (p.Arg25*), known *EDA* mutation c.491A>C (p.Glu164Ala), and known *Wnt family member 10A* (*WNT10A*) mutations c.511C>T (p.Arg171Cys) and c.742C>T (p.Arg248*) were identified in families with NSTA. The novel *EDA* and *EDAR* mutations were predicted as being pathogenic through bioinformatics analyses and structural modeling. Two variants of *WNT10A*, c.374G>A (p.Arg125Lys) and c.125A>G (p.Asn42Ser), were found in patients with NSTA. The two *WNT10A* variants were predicted to affect the splicing of message RNA, but minigene experiments showed normal splicing of mutated minigenes. This study uncovered the genetic foundations with respect to six families with XLHED or NSTA. We identified six mutations, of which two were novel mutations of *EDA* and *EDAR*. This is the first report of a nonsense *EDAR* mutation leading to NSTA.

## 1. Introduction

Tooth agenesis (TA) is a disorder characterized by congenital tooth loss. According to a meta-analysis, the mean prevalence of TA is 6.53 ± 3.33% [1]. The high prevalence of TA, as well as the esthetic and functional problems it causes, make it a significant dental issue [2]. There are two sub-types of TA: non-syndromic TA (NSTA) and syndromic TA (STA). In NSTA, the patients do not have anomalies other than congenital tooth loss [3]. In STA, tooth agenesis is a symptom of a syndrome [4]. 

NSTA is a disorder with great genetic heterogeneity. Many genes have been reported as being associated with NSTA. *Wnt family member 10A* (*WNT10A*) is a key mediator of WNT signaling. Suppression of WNT signaling arrests tooth development [5]. Yamashiro et al. reported that Wnt10a may have a link with the differentiation of odontoblasts and cusp morphogenesis [6]. Zhu et al. found that WNT/β-catenin activity is required for tooth development at the early cap stage [7]. In recent years, *WNT10A* was found to be mutated in 28–62% of patients with NSTA [8,9,10]. Tardieu et al. found that heterozygous *WNT10A* mutations resulted in relatively fewer missing teeth, while compound heterozygous *WNT10A* mutations caused more missing teeth [11]. In addition to WNT signaling, EDA/EDAR/EDARADD signaling has been shown to play a significant role in NSTA [12,13]. Tucker et al. showed that Edar/Eda interactions regulate enamel knot formation in tooth morphogenesis [14]. *MSX1* and *PAX9* play an important role in tooth development. In *Pax9^−^*^/*−*^ or *Msx1^−^*^/*−*^ mice, tooth development is arrested in the bud stage [15,16]. This is because the two genes can synergistically activate the *BMP4* gene, which is vital for the evolution from the bud to cap stage in tooth budding [17]. Mutations of *MSX1* and *PAX9* account for a proportion of NSTA cases [18,19]. Mutations of some other genes, such as *AXIN2*, *WNT10B*, *LRP6*, *EDAR*, and *EDARADD*, have also been identified in a few patients [19,20].

STA is comprised of many diseases, including hypohidrotic ectodermal dysplasia (HED) and odonto-onycho-dermal dysplasia (OODD). Hypohidrotic ectodermal dysplasia is characterized by sparse hair, oligodontia, and reduced sweating [21]. The inheritance of HED can be X-linked, autosomal dominant, or autosomal recessive [22]. X-linked HED (XLHED) is caused by mutations of *Ectodysplasin A* (*EDA*) [23,24]. Mutations of both *EDA receptor* (*EDAR*) and EDAR-associated death domain (*EDARADD*) genes are reported to cause autosomal-dominant (ADHED) and autosomal-recessive HED (ARHED) [25,26]. Cluzeau et al. and our group reported that *WNT10A* also mutated in some patients with ADHED or ARHED [24,27]. Odonto-onycho-dermal dysplasia (OODD), another kind of STA characterized by massive loss of teeth, hyperhidrosis, palmoplantar hyperkeratosis, and nail dystrophy, is caused by mutations of *WNT10A* [28].

From our previous introduction, we know that, although NSTA and STA are two different subtypes of tooth agenesis, they can be caused by mutations of the same genes. *EDA*, *EDAR*, *EDARADD*, and *WNT10A* are candidate genes of both NSTA and STA. In our previous study, we found that patients with XLHED suffer only from oligodontia and sparse hair, representing a middle stage between XLHED and NSTA [29]. Such patients presenting with a middle status between XLHED and NSTA have been reported by other researchers as well [30,31]. We proposed that XLHED and *EDA*-related NSTA are possibly the same disease, caused by *EDA* mutations but with different degrees of severity [29]. Bohring et al. and Vink et al. reported that *WNT10A* mutations can cause a broad spectrum of phenotypic variability, from OODD to NSTA [32,33]. The phenotypic variability exists at both inter- and intra-familial levels [33]. We hypothesize that some cases of STA and NSTA, caused by mutations of the same gene, represent the same disease but with phenotypic variability.

In this study, to further investigate the genetic basis and relationship of STA and NSTA, we studied genetic defects in two families with XLHED and four families with NSTA through Sanger sequencing, whole exome sequencing, and bioinformatics analyses. Two novel and four known pathogenic mutations were identified.

## 2. Materials and Methods 

### 2.1. Subjects

The study aimed to uncover the genetic basis and relationship of XLHED and NSTA. The study was approved by the Ethical Review Committee at the Guanghua School and Hospital of Stomatology at Sun Yat-Sen University (ERC-[2013]-9, date of approval: 1 December 2013, and ERC-[2014]-25, date of approval: 1 November 2014). Recommendations from the Declaration of Helsinki were followed. We obtained informed consent from all of the participants. The inclusion criterion for XLHED was the presence of at least two out of the three following symptoms: sparse hair, oligodontia, and reduced sweating [27]. The patient was excluded if the diagnosis of another kind of ectodermal dysplasia was made [22]. The inclusion criterion of NSTA was the presence of congenital loss of teeth. The exclusion criterion of NSTA was the involvement of other symptoms, such as hypohidrosis, hypotrichosis, and cleft lip and palate. In this project, all of the participants were assessed clinically except the uncle of the proband in XLHED1. Two patients with XLHED, four patients with NSTA, and their family members were recruited, and 4 mL of peripheral blood were collected for each participant at the Department of Pediatric Dentistry, Guanghua Hospital of Stomatology. The uncle of the proband of XLHED Family 1 lives in another city. Therefore, his blood sample was collected in a local hospital and mailed to us.

### 2.2. Mutation Detection of WNT10A, EDA, EDAR, EDARADD, PAX9, MSX1, AXIN2, LRP6, and WNT10B

We extracted genomic DNA from the blood. Primers covering coding and flanking sequences of *WNT10A*, *EDA*, *EDAR*, and *EDARADD* were reported in our previous studies [24,29]. We designed primers to amplify *PAX9*, *MSX1*, *AXIN2*, *LRP6*, and *WNT10B.* The primer sequences are listed in Tables S1–S5. We then performed Polymerase Chain Reaction (PCR) in order to amplify the exons of nine genes, and the products were sequenced on an ABI 3730XL genetic analyzer (Applied Biosystems, Foster City, CA, USA). Mutation nomenclature was used, with +1 corresponding to the A of the ATG translation initiation codon of the reference sequence NM_025216.2 (*WNT10A*), NM_001399.4 (*EDA*). The population genetics data from 1000 Genomes (http://browser.1000genomes.org/index.html) and Database of Single Nucleotide Polymorphisms (dbSNP, https://www.ncbi.nlm.nih.gov/snp) were used as control to rule out population polymorphism.

### 2.3. Whole Exome Sequencing and Data Analysis

Whole exome sequencing was performed for the proband of Family 4, who did not have any mutations detected with respect to *WNT10A*, *EDA*, *PAX9*, and *MSX1*. Whole exome sequences were enriched with xGen^®^ Exome Research Panel v1.0 (Integrated DNA Technologies, Coralville, IA, USA). The enriched samples were sequenced with an Illumina HiSeq 2000 to generate 150 bp paired-end reads. Reads were aligned to the human reference genome hg19 using the Burrows–Wheeler Aligner [34]. Single Nucleotide Polymorphisms (SNPs) and Insertions and Deletions (InDels) were identified by the Genome Analysis Toolkit (GATK) and annotated by ANNOVAR [35,36]. Variants of *EDAR*, *EDARADD*, *AXIN2*, *WNT10B*, *LRP6*, and *LTBP3* genes were extracted and filtered with the criteria of “minor allele frequency (MAF) < 1%” and “exonic”. The candidate mutation of *EDAR* was verified with PCR, followed by Sanger sequencing. PCR was performed, and the PCR products were sequenced as described in Section 2.2. The reference sequence for *EDAR* was NM_022336.3.

### 2.4. Bioinformatics Analyses

Domain information of EDA, WNT10A, and EDAR was retrieved from the Human Protein Reference Database (http://hprd.org/query). PolyPhen 2 [37], Sorting Intolerant from Tolerant (SIFT) [38], Mutation Taster [39], and Human Splicing Finder [40] were used to predict the effect of novel missense mutation. CLUSTAL X (1.83) [41] was used to compare the human wild-type WNT10A protein (CCDS2426.1) with those of the chimpanzee (ENSPTRT00000023964.3), dog (ENSCAFT00000023775.4), rat (NP_001101697), mouse (CCDS15057), chicken (NP_001006590), and zebrafish (NP_571055). A cross-species alignment of EDA protein was performed as described in a previous study [29].

### 2.5. Structural Modeling

The structures of wild-type and mutated EDA and WNT10A proteins were modeled with Swiss Pdb Viewer v4.1 [42]. The structure of EDA (PDB ID 1RJ7; X-ray, resolution 2.3Å) and Wnt-8 (PDB ID 4F0A; X-ray, resolution 3.25Å) was used as templates for homology modeling. Visualization of the three-dimensional (3D) structures were performed with PyMol v1.5.0.3 (The PyMOL Molecular Graphics System, Version 1.8 Schrödinger, LLC., Cambridge, MA, USA).

### 2.6. Minigene Study

A minigene was designed to analyze whether *WNT10A* variants affected the splicing of message RNA. Exon 1, Intron 1, Exon 2, Partial Intron 2 (containing the first 203 bp and the last 210 bp) and Exon 3 of *WNT10A* were amplified by PCR from the genomic DNA of the probands of NSTA Families 1 and 3, who carried the heterozygous *WNT10A* variants c.374G>A (p.Arg125Lys) and c.125A>G (p.Asn42Ser). The primers were as follows: E1E2-F: 5′-ACTTAAGCTTGCCCGTCAGGGCCTGCGCG-3′; E1E2-R: 5′-**AGGCTTCCCACCAGTCTGGAGA**CACATTTG-3′; E3-F: 5′-**TCTCCAGACTGGTGGGAAGCCT**CCCTCCCA-3′; E3-R: 5′-GCCCTCTAGATGAAGCAGACCCAGGGGTGG-3′. The underlined sequences are restriction endonuclease sites for *Hind*III and *Xba*I. The sequences in bold are homologous sequences for overlap PCR. The PCR products of E1E2 and E3 were assembled together by overlap PCR with E1E2-F and E3-R.

The PCR products of E1E2-F and E3-R were digested and ligated to the pcDNA3.1(+) vector (Invitrogen Corporation, Carlsbad, CA, USA) at the *Hind*III and *Xba*I cloning sites. The plasmid constructs containing wild-type, c.374G>A, or c.125A>G variants were examined by PCR and Sanger sequencing. The primers were as follows: pcDNA-F: 5′-CGCAAATGGGCGGTAGGCGTG-3′; WNT10A-F: 5′-CAGGAGTAAGCACAAGCTG-3′; WNT10A-R: 5′-GGGAGGGAGGCTTCCCACCAGTCTGGAGACACATTTG-3′; pcDNA-R: 5′-CCAGGGTCAAGGAAGGCACG-3′.

HEK-293T and HeLa cells were cultured in Dulbecco’s modified Eagle’s medium (DMEM) containing 10% fetal bovine serum at 37 °C, with 5% CO_2_. Two micrograms of pcDNA3.1(+) negative control vector, wild-type minigene construct, c.374G>A minigene construct, and c.125A>G minigene construct were transfected into HEK-293T and HeLa cells with Lipofectamine^TM^ 2000 (Invitrogen, CA, USA). 

At 48 h after transfection, total RNA was extracted with Trizol reagents (Invitrogen, CA, USA). Reverse transcription was performed with PrimeScript™RT reagent Kit (TaKaRa, Beijing, China). First strand complementary DNA (cDNA) was amplified by PCR and followed by Sanger sequencing. The primers were as follows: forward: 5′-CGTTTAAACTTAAGCTTGCCCGTCAGGGCCTGCGCG-3′; reverse: 5′-TAGAAGGCACAGTCGAGG-3′.

## 3. Results

### 3.1. Clinical Report 

**Figure 1:**
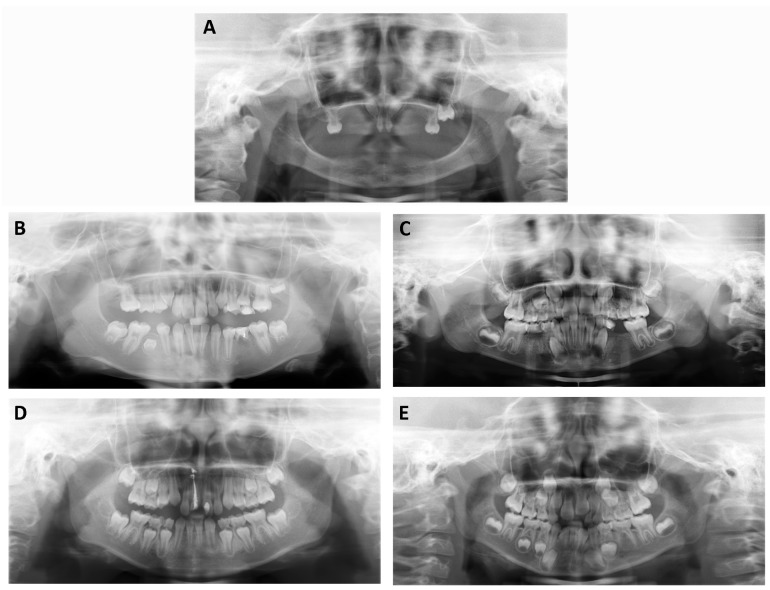
Panoramic radiograph of the probands of X-linked hypohidrotic ectodermal dysplasia (XLHED) Family 2 (**A**); non-syndromic tooth agenesis (NSTA) Family 1 (**B**); NSTA Family 2 (**C**); NSTA Family 3 (**D**); and NSTA Family 4 (**E**). They were missing 23, 4, 9, 2, and 6 permanent teeth, respectively. Because the proband of XLHED1 was too young to cooperate, we were not able to assess the missing teeth of the patient by panoramic radiograph. For the uncompressed Figure 1 file, please download the Supplementary Materials.

The proband (III:1, Table 1) of XLHED Family 1 was a two-year-old boy with no history of eruption of primary teeth. We attempted to assess the missing teeth of the patient by panoramic radiograph, but we failed because the patient was too young to cooperate. His hair and eyebrows were sparse. He was hypohidrotic and presented with intolerance to heat. The uncle (II:3) of the proband had 22 missing permanent teeth, as he himself reported. He was an adult without a history of tooth extraction. His teeth were congenitally missing. He also suffered from hypohidrosis and hypotrichosis. A 6-year-old boy (II:1, Table 1) was the only patient of XLHED Family 2. Panoramic radiograph showed that he had 18 missing primary teeth and 23 missing permanent teeth (excluding the third molars) (Figure 1A, Table 2). The shape of the incisors was conic. He was hypohidrotic, and his hair was sparse.

**Figure 2:**
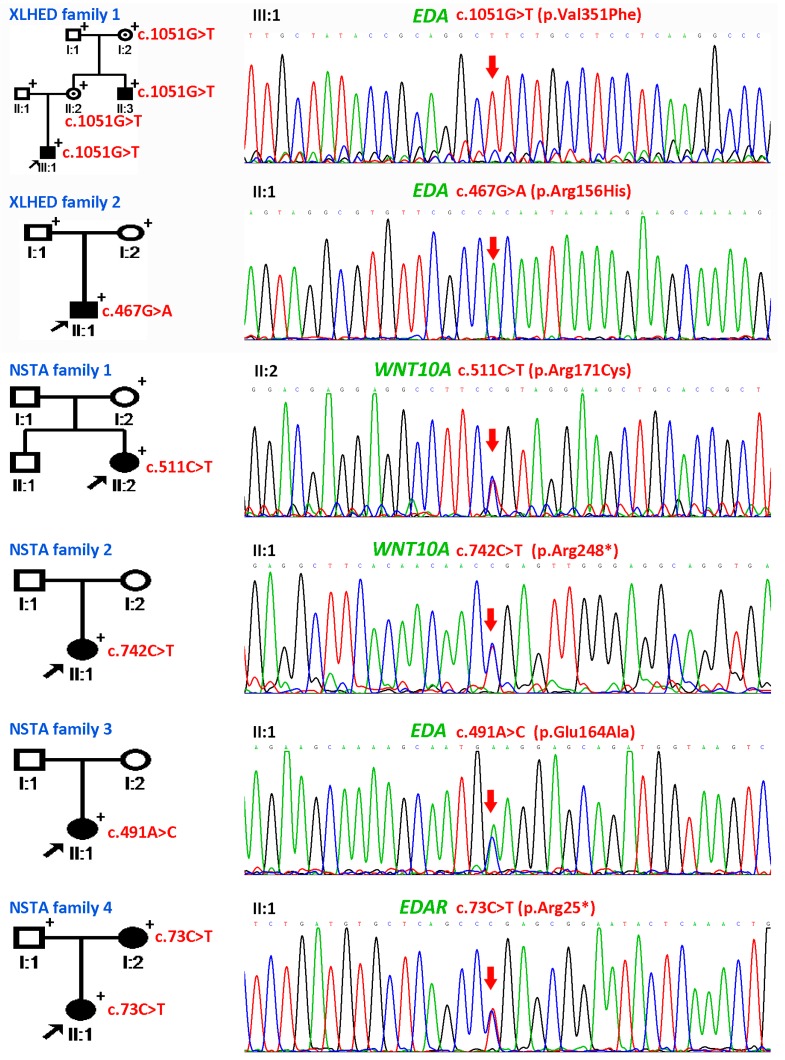
Pedigrees and mutation information of X-linked hypohidrotic ectodermal dysplasia (XLHED) Families 1–2 and non-syndromic tooth agenesis (NSTA) Families 1–4. In XLHED Family 1, a novel *Ectodysplasin A* (*EDA*) mutation c.1051G>T (p.Val351Phe) was cosegregated in the family. In XLHED Family 2, the known de novo *EDA* mutation c.467G>A (p.Arg156His) was identified in the genome of the proband. In NSTA Family 1, the proband carried the heterozygous *Wnt family member 10A* (*WNT10A*) mutation c.511C>T (p.Arg171Cys). In NSTA Family 2, the heterozygous *WNT10A* mutation c.742C>T (p.Arg248*) was identified in the genome of the proband. In NSTA Family 3, the proband was found to have the heterozygous *EDA* mutation c.491A>C (p.Glu164Ala). In NSTA Family 4, the proband and her mother shared the *EDA receptor* (*EDAR*) mutation c.73C>T (p.Arg25*), while her father was wild-type at this location. Black arrows pointed to the probands. The individuals, whose blood samples were available, are marked by “+”. Red arrows indicate the mutations.

The probands of the four NSTA families presented with congenital loss of teeth, without any other accompanying symptoms. The shape of the teeth was normal. The numbers of missing permanent teeth are listed in Table 1. The diagnosis of tooth agenesis was confirmed by panoramic radiographs (Figure 1B–E, Table 2). We did not evaluate the number of missing primary teeth of the patients because some primary teeth may be replaced by permanent teeth. The pedigrees are reported in Figure 2. There was only one patient per family in NSTA Families 1–3. In NSTA Family 4, the mother of the proband had six congenitally missing teeth.

### 3.2. Genetic Findings of WNT10A, EDA, PAX9, and MSX1

**Figure 3:**
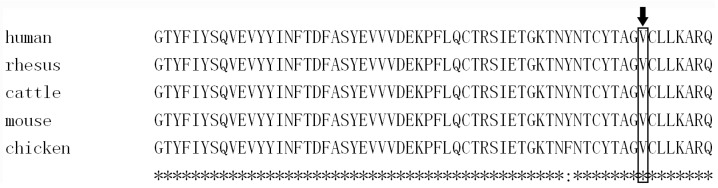
A cross-species alignment of amino acid sequence of Ectodysplasin A (EDA) showed that p.Val351 was conserved.

**Figure 4:**
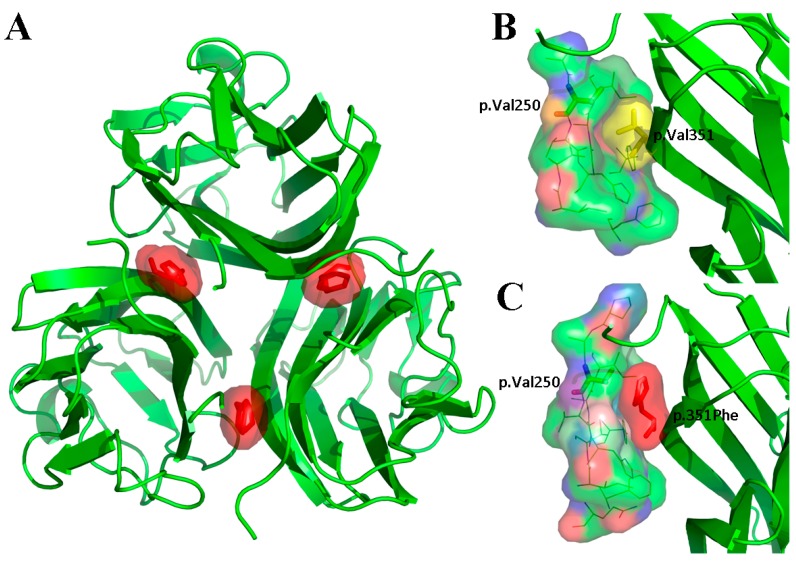
Structural modeling results of the Tumour Necrosis Factor (TNF) homology domain of p.Val351Phe-mutated (**A**, **C**) and wild-type (**B**) Ectodysplasin A (EDA) proteins. (**A**) The p.Val351Phe mutation was located on the monomer–monomer interaction surface of the three monomers. (**B**) In the wild-type EDA protein, there was a small gap between p.Val351 and p.Val250 of the adjacent monomer. (**C**) In the p.Val351Phe-mutated EDA protein, the mutated p.351Phe was too large and extruded with p.Val250 of the adjacent monomer.

Sequencing of the *EDA* gene in XLHED Family 1 revealed novel mutation c.1051G>T (p.Val351Phe) shared by the proband and his uncle (Figure 2). His mother and grandmother were asymptomatic heterozygous carriers of the mutation. His father and grandfather were wild-type at c.1051. The mutation was located at the Tumour Necrosis Factor (TNF) homology domain of the EDA protein, and replaced a valine with a phenylalanine at amino acid 351. The mutation was not reported by dbSNP, 1000 Genomes, the Human Gene Mutation Database (public version), or PubMed. A cross-species alignment of the EDA protein showed that p.Val351 was conserved (Figure 3A). SIFT, PolyPhen2, and the Mutation Taster predicted the mutation as being “damaging”, “probably damaging”, and “disease causing”, respectively. Structural modeling of the p.Val351Phe-mutated TNF homology domain of the EDA protein showed that the mutation was located on the surface of the three monomers (Figure 4A). Compared with the wild-type p.Val351 (Figure 4B), the mutated p.351Phe was too large and extruded with p.Val250 of the adjacent monomer (Figure 4C). 

Screening of *EDA* in XLHED Family 2 showed a known de novo mutation c.467G>A (p.Arg156His) in the genome of the patient (Figure 2). The parents of the patient were normal and did not carry the mutation. The mutation c.467G>A is one of the most prevalent mutations found in patients with XLHED [43].

We screened for mutation of *WNT10A* and *EDA* genes in the probands of four NSTA families. In the genome of the proband of NSTA Family 1, the known mutation c.511C>T (p.Arg171Cys) and the variant c.374G>A (p.Arg125Lys) were identified in the *WNT10A* gene (Figure 2). The mother of the proband carried a c.374G>A variant but was free of the c.511C>T mutation. In Family 2, the known heterozygous *WNT10A* mutation c.742C>T (p.Arg248*) was identified in the genome of the proband (Figure 2). Blood samples of the parents were not available. In Family 3, we found the known heterozygous *EDA* mutation c.491A>C (p.Glu164Ala) and the heterozygous *WNT10A* variant c.125A>G (p.Asn42Ser, rs149865858) in the genome of the proband (Figure 2). The parents of the proband were not willing to participate in this study, so we were not able to analyze the genotype of the parents. 

Because we did not identify any mutations of *WNT10A* and *EDA* genes in Family 4, we performed Sanger sequencing for the *PAX9* and *MSX1* genes. The proband of Family 4 was negative for *PAX9* and *MSX1* mutation. 

### 3.3. Results of Whole Exome Sequencing

**Figure 5:**
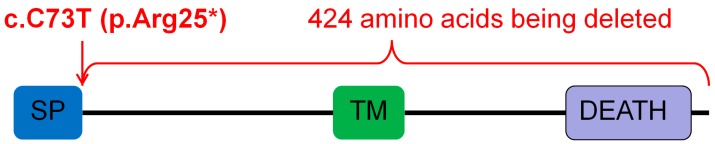
*Ectodysplasin A receptor* (*EDAR*) mutation c.73C>T (p.Arg25*) introduced a stop codon at amino acid 25 of the encoded protein, truncating 424 amino acids of the protein. SP: signal peptide; TM: transmembrane domain; DEATH: death domain.

Subsequently, the proband of Family 4 was assigned for whole exome sequencing. In total, 27,013,053 reads were obtained. The average coverage of the target sequences was 64.18X. All in all, 94.9% of the target sequences were covered with at least 20X. There were 64,338 SNPs and 9797 indels identified. The variants of *EDAR*, *EDARADD*, *AXIN2*, *WNT10B*, *LRP6*, and *LTBP3* genes were called, and we obtained 43 SNPs and 3 indels. After filtering with “minor allele frequency (MAF) < 1%” and “exonic”, we obtained the novel *EDAR* mutation c.73C>T (p.Arg25*). This mutation was confirmed by Sanger sequencing. The mother of the proband carried the mutation, while the father was wild-type at the locus (Figure 2). The mutation induced a termination codon at the amino acid 25 of the encoded protein. The mutated protein was unlikely to have any function as the majority of the protein was truncated (Figure 5). This mutation was not reported in 1000 Genomes, the Human Gene Mutation Database (HGMD; public version), or PubMed. 

### 3.4. Bioinformatics Analyses and Minigene Results of WNT10A Variants c.374G>A and c.125A>G

We identified *WNT10A* variants c.374G>A in NSTA Family 1, and c.125A>G in NSTA Family 3, as described in Section 3.2. Some analyses and experiments were done to find out whether they were pathogenic or not.

**Figure 6:**
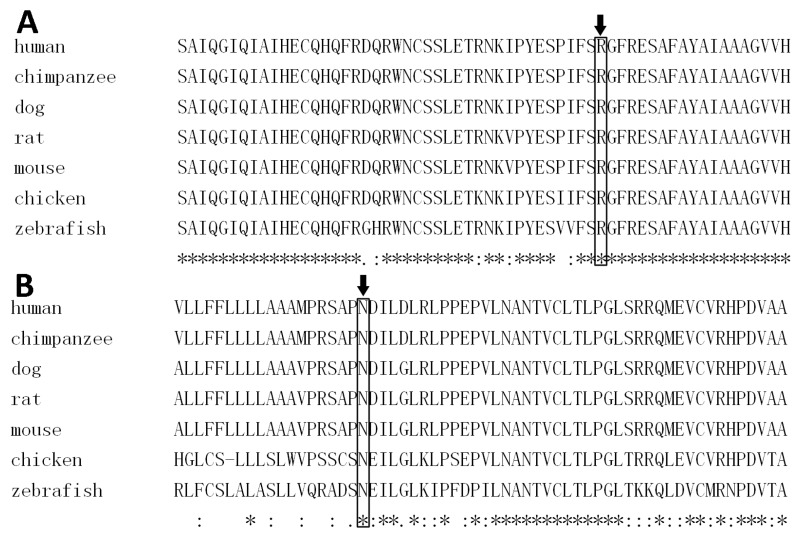
A cross-species alignment of amino acid sequence of Wnt family member 10A (WNT10A) indicated that p.Arg125 (**A**) and p.Asn42 (**B**) were conserved.

**Figure 7:**
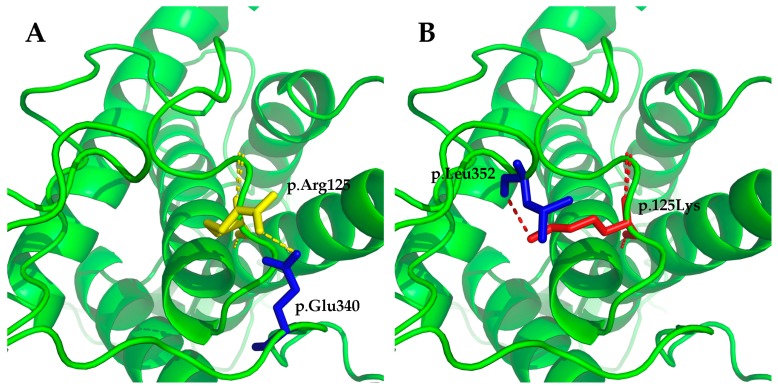
Structural modeling results of the wild-type Wnt family member 10A (WNT10A) protein and the p.Arg125Lys-mutated WNT10A protein. (**A**) There was a hydrogen bond between p.Arg125 and p.Glu340 in the wild-type WNT10A protein. (**B**) The original hydrogen bond vanished in the p.Arg125Lys-mutated WNT10A protein, and a new hydrogen bond between p.125Lys and p.Leu352 arose. The changes may lead to instability of the structure and affect the affinity to the receptor of WNT10A.

The *WNT10A* variant c.374G>A (p.Arg125Lys) was not reported in 1000 Genomes, the Human Gene Mutation Database (HGMD; public version), or PubMed. A cross-species alignment of the amino acid sequence of WNT10A showed that p.Arg125 was conserved (Figure 6A). The variant led to a substitution of an arginine to a lysine at the position. It was predicted as being “benign” by PolyPhen 2, and “tolerated” by SIFT. Mutation Taster predicted the mutation as being “disease causing” because it changed the amino acid sequence, and it may disrupt the splicing of the second exon with the third exon. Human Splicing Finder (HSF) also predicted that the mutation could affect the splicing of mRNA by creating a new exonic splicing silencer and breaking down an exonic splicing enhancer. Structural modeling results showed that there was a hydrogen bond between p.Arg125 and p.Glu340 in the wild-type WNT10A protein (Figure 7A). This hydrogen bond vanished in the p.Arg125Lys-mutated WNT10A protein, and a new hydrogen bond between p.125Lys and p.Leu352 arose (Figure 7B). 

The c.125A>G mutation occurred at the evolutionary conserved p.Asn42 (Figure 6B) and changed the amino acid to serine. It was predicted to be “tolerated” by SIFT. However, PolyPhen 2 predicted it as being “probably damaging”, and Mutation Taster predicted it as being “disease causing”. The splicing of Exons 1 and 2 may be affected by the mutation, predicted by Mutation Taster and HSF. This mutation was not reported by the HGMD public version or PubMed, but the 1000 Genomes database recorded three heterozygous allele carriers of the *WNT10A* mutation c.125A>G. Structural modeling for the p.Asn42Ser-mutated WNT10A protein was not feasible due to the fact that the homologous template 4F0A does not cover p.Asn42. 

**Figure 8:**
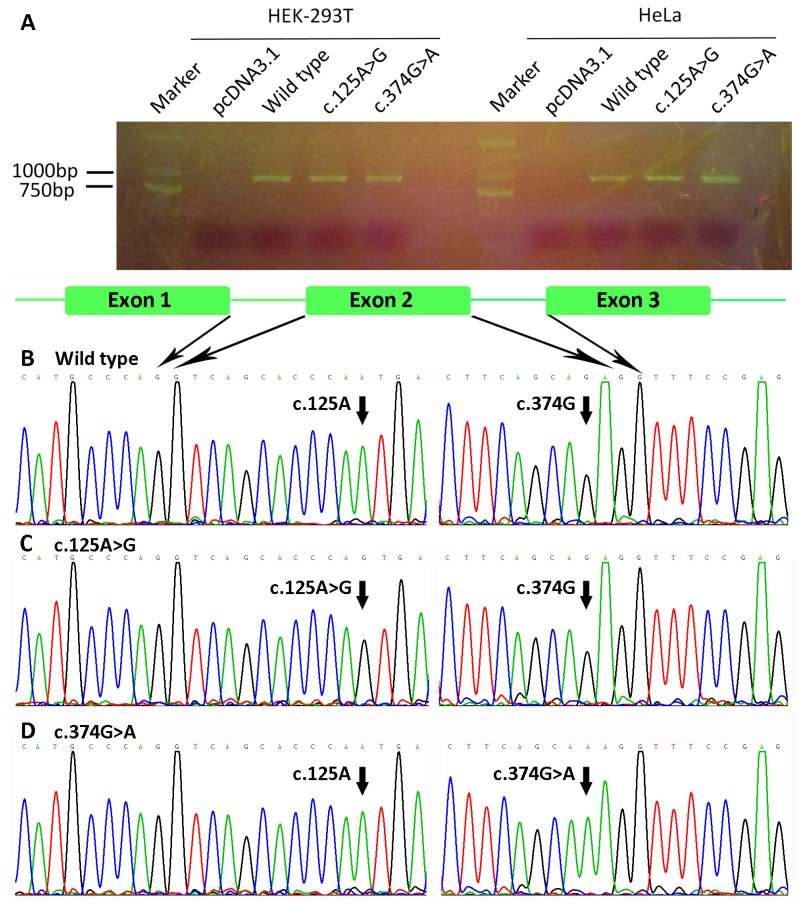
Minigene splicing assay of *Wnt family member 10A* (*WNT10A*) variants c.374G>A and c.125A>G. (**A**) Agarose gel electrophoresis of PCR products. No abnormal splicing product was found in either HeLa or HEK-293T cells. (**B**–**D**) Sequencing results of (**B**) the wild-type minigene, (**C**) the c.125A>G minigene, and (**D**) the c.374G>A minigene. The minigenes containing variant c.374G>A or c.125A>G spliced normally.

A minigene splicing assay was used to analyze the mRNA-splicing effect of *WNT10A* variants c.374G>A and c.125A>G. The minigenes, containing wild-type, c.374G>A, or c.125A>G of *WNT10A* Exons 1–3, were cloned into the pcDNA3.1 plasmid. HeLa and HEK-293T cells were used to investigate the splicing effect of the variants. As shown in Figure 8A, no abnormal splicing product was found. Sequencing results of the splicing products showed normal splicing of the two minigenes, which contained variant c.374G>A or c.125A>G (Figure 8B–D).

### 3.5. Sequencing Results of PAX9, MSX1, EDAR, EDARADD, AXIN2, LRP6, and WNT10B in NSTA Families 1 and 3 

To rule out the possibility of mutations in other NSTA candidate genes, we sequenced *PAX9*, *MSX1*, *EDAR*, *EDARADD*, *AXIN2*, *LRP6*, and *WNT10B* genes of the probands in NSTA Families 1 and 3. No pathogenic mutation was found.

## 4. Discussion

This article represented a comprehensive genetic study of XLHED and NSTA. Sanger sequencing of nine candidate genes and whole exome sequencing were performed, and the genetic defects of six families with XLHED or NSTA were identified. *EDA* mutation c.1051G>T (p.Val351Phe) and *EDAR* mutation c.73C>T (p.Arg25*) were novel. Bioinformatics analyses and structural modeling were used to evaluate the pathogenicity of the mutations. A minigene splicing assay was performed, and the results showed that *WNT10A* variants c.374G>A and c.125A>G did not affect the splicing of mRNA.

Mues et al. revealed that NSTA-related *EDA* mutations only impaired the receptor binding capability of mutant EDA proteins, while STA-related *EDA* mutations abolish the receptor binding capability of mutant EDA proteins [44]. This supports that theory that the development of the human dentition requires the highest level of EDA-receptor signaling, whereas other ectodermal appendages have less stringent requirements for EDA-receptor signaling [44]. In a previous study, we identified the *EDA* mutation c.776C>A (p.Ala259Glu) in a patient with XLHED [29]. Interestingly, the same mutation was also identified in patients with NSTA [45]. We proposed that that XLHED and EDA-related NTA are the same disease with different degrees of severity [29]. In this study, we found the heterozygous *WNT10A* mutation c.742C>T (p.Arg248*) in NSTA Family 2. This mutation in homozygous status also was reported in a patient with STA (HED) [27]. Vink et al. reported compound heterozygous *WNT10A* mutations c.682T>A (p.Phe228Ile) and c.321C>A (p.Cys107*) in a patient with STA (OODD), while the patient’s mother carried heterozygous *WNT10A* mutations c.682T>A (p.Phe228Ile) and suffered from NSTA [33]. The data indicated that *WNT10A*-related STA (HED or OODD) and *WNT10A*-related NSTA are the same disease with phenotypic variability. Based on the data of our group and the results of other researchers, we think some cases of STA and NSTA, which are caused by mutations of the same gene, are examples of the same disease with phenotypic variability. We predict that there will be more cases of the same mutations found in both patients with STA and NSTA.

In most of the previous studies, *EDAR* mutations were found to be the cause of hypohidrotic ectodermal dysplasia [27,46,47]. There have only been two works reporting *EDAR* mutations in patients with NSTA [12,48]. However, all of the *EDAR* mutations in these two studies were missense mutations. In our study, a novel nonsense *EDAR* mutation c.73C>T (p.Arg25*) was identified in NSTA Family 4. This mutation truncated 424 amino acids of the EDAR protein. The mutated protein lost its transmembrane domain and death domain. Therefore, the mutated protein is not expected to have any function. This is the first study of a nonsense *EDAR* mutation leading to the onset of NSTA.

There are three functional domains of the EDA protein, namely a furin protease recognition sequence, a collagen domain, and a C-terminal TNF homology domain [49]. The TNF homology domain forms homotrimers, which can bind with EDAR [50]. In this study, the novel mutation c.1051G>T (p.Val351Phe) was located on the surface of monomers. Structural modeling results showed that the mutation probably hampers the monomer–monomer interaction. The trimer may become globally instable. [51] Additionally, because the monomer–monomer interface is the core region for EDA–EDAR contact and signal transduction, the mutated EDA protein may lose its specificity to EDAR. [51] Bioinformatics analyses results, including software prediction, amino acid conservation analysis, and a public population genetic database, also indicated the deleterious nature of the mutation. 

In NSTA Families 1 and 3, apart from the mutations in *EDA* (c.491A>C) and *WNT10A* (c.511C>T), we also found *WNT10A* variants c.374G>A and c.125A>G. The *WNT10A* mutation c.511C>T (p.Arg171Cys) was a pathogenic mutation reported in patients with tooth agenesis [52]. The *EDA* mutation c.491A>C (p.Glu164Ala) in NSTA Family 3 was an established pathogenic mutation, which is responsible for hypodontia and X-linked hypohidrotic ectodermal dysplasia [53]. Results of prediction using some software (SIFT and PolyPhen 2) did not support that the two *WNT10A* variants (c.374G>A and c.125A>G) were pathogenic. However, the Mutation Taster and Human Splicing Finder predicted that the two variants may affect the splicing of mRNA. Hence, minigene splicing experiments were performed. The results showed that the minigenes containing *WNT10A* variants c.374G>A or c.125A>G can splice normally. Hence, we consider that the two *WNT10A* variants were not pathogenic and did not play a role in the onset of the disease. The patient of NSTA Family 1 is caused by *WNT10A* mutation c.511C>T (p.Arg171Cys), and the patient of NSTA Family 3 is caused by *EDA* mutation c.491A>C (p.Glu164Ala).

There are 10 known causal genes of NSTA (*WNT10A*, *EDA*, *PAX9*, *MSX1*, *AXIN2*, *EDAR*, *EDARADD*, *WNT10B*, *LRP6*, and *LTBP3*) [10,12,17,20]. Screening all of the candidate genes for pathogenic mutations by Sanger sequencing would imply high financial costs and time requirements. Hence, we performed the experiments in a more efficient and economical way. In the first step, we screened for two of the most frequently mutated genes (*WNT10A* and *EDA*). If no pathogenic mutation was found, then we screened for two additional frequently mutated genes (*PAX9* and *MSX1*). If still no pathogenic mutation was found, we applied whole exome sequencing to detect mutations of other six candidate genes. This strategy worked in the four probands. Two of the probands had mutations in the *WNT10A* gene, one of the probands had a mutation in the *EDA* gene, and the last proband had a mutation in the *EDAR* gene. To verify our findings and rule out the possibility of mutations in undetected genes, we screened seven more known candidate genes (*PAX9*, *MSX1*, *AXIN2*, *EDAR*, *EDARADD*, *WNT10B*, and *LRP6*) in NSTA Families 1 and 3. We did not find any pathogenic mutation in the exons and flanking regions. We did not check for mutations in *LTBP3* because patients with *LTBP3* mutations are of short stature [54], and the patients in this study were of normal stature. We did not screen the seven genes in NSTA Family 2 because the patient had a nonsense *WNT10A* mutation c.742C>T (p.Arg248*), which is pathogenic through the truncation of the C-terminal of the WNT10A protein.

## 5. Conclusions

In this study, we identified three *EDA*, one *EDAR* and two *WNT10A* mutations in two patients with X-linked hypohidrotic ectodermal dysplasia (XLHED) and four patients with non-syndromic tooth agenesis (NSTA). *EDA* mutation c.1051G>T (p.Val351Phe) and *EDAR* mutation c.73C>T (p.Arg25*) were novel. For the first time, these two novel mutations are hereby proven to be pathogenic. We have here reported the first nonsense *EDAR* mutation leading to the onset of NSTA. These results expand the mutational spectrum of *WNT10A* and *EDAR* genes and contribute to the practice of precision medicine. 

## Figures and Tables

**Table 1:** Summary of clinical data and mutations in *EDA*, *EDAR*, and *WNT10A* genes.

Family	Patient	Age and Gender	Gene	Nucleotide Change	Amino Acid Change	Number of Missing Permanent Teeth ^#^
XLHED1	III:1	2y, M	*EDA*	c.1051G>T	p.Val351Phe	ND
XLHED2	II:1	6y, M	*EDA*	c.467G>A	p.Arg156His	23
NSTA1	II:2	10y, F	*WNT10A*	c.511C>T	p.Arg171Cys	4
NSTA2	II:1	6y, F	*WNT10A*	c.742C>T	p.Arg248*	9
NSTA3	II:1	9y, F	*EDA*	c.491A>C	p.Glu164Ala	2
NSTA4	II:1	6y, F	*EDAR*	c.73C>T	p.Arg25*	6

^#^ Excluding the third molars. Bold type: novel mutation; ND: not determined; XLHED: X-linked hypohidrotic ectodermal dysplasia; NSTA: non-syndromic tooth agenesis; *EDA*: *Ectodysplasin A*; *EDAR*: *EDA receptor*; *WNT10A*: *Wnt family member 10A*.

**Table 2:** Summary of missing permanent teeth of the five probands whose panoramic radiographs are available.

Family	Patient	MT ^#^		Right								Left							
				8	7	6	5	4	3	2	1	1	2	3	4	5	6	7	8
**XLHED2**	II:1	23	Maxillary	*	*		*	*	*	*			*	*	*	*			*
			Mandibular	*	*	*	*	*	*	*	*	*	*	*	*	*	*	*	*
**NSTA1**	II:2	4	Maxillary	*	*														*
			Mandibular	*						*			*			*			*
**NSTA2**	II:1	9	Maxillary	*			*			*			*		*	*			*
			Mandibular	*			*	*							*	*			*
**NSTA3**	II:1	2	Maxillary	*															*
			Mandibular	*						*			*						*
**NSTA4**	II:1	6	Maxillary	*			*						*			*			*
			Mandibular	*							*	*				*			*

^#^ MT: number of missing permanent teeth, excluding the third molars; * indicates missing teeth; XLHED: X-linked hypohidrotic ectodermal dysplasia; NSTA: non-syndromic tooth agenesis.

## Supplementary Materials

The following are available online at www.mdpi.com/2073-4425/8/10/259/s1. Table S1. Primers amplifying coding exons and flanking intronic sequences of *PAX9*; Table S2. Primers amplifying coding exons and flanking intronic sequences of *MSX1*; Table S3. Primers amplifying coding exons and flanking intronic sequences of *AXIN2*; Table S4. Primers amplifying coding exons and flanking intronic sequences of *LRP6*; Table S5. Primers amplifying coding exons and flanking intronic sequences of *WNT10B*.
