# Supplementary material for: Novel EDA or EDAR Mutations Identified in Patients with X-Linked Hypohidrotic Ectodermal Dysplasia or Non-Syndromic Tooth Agenesis
Source: Genes (Basel). 2017 Oct 5;8(10):259. doi: 10.3390/genes8100259 (PMC5664109; doi:10.3390/genes8100259)
Supplement: Supplementary file 1 [file genes-08-00259-s001.zip › Supplementary Materials/Table S1-5 primers and Figures.docx]

Table S1. Primers amplifying coding exons and flanking intronic sequences of *PAX9*

| **Exon** | **Forward (5’-3’)** | **Reverse (5’-3’)** |
| --- | --- | --- |
| **1** | CCACGTTGCTGCTTAGATTGAAAT | GGAACACGAGCAAAGTCAATAGAG |
| **2** | TTCGGCTATGTTCAGGGACCATAT | GGTGGTGGGAAAGACAGTGTC |
| **3** | CTCTGAGGGGAGTAAAACTTCACC | CACGAAGGATCTGGCTCGTAG |
| **4** | TTGGAGAGTAGAGTCAGAGCATTG | GTGAGACCTGGGAATTGGGG |

Table S2. Primers amplifying coding exons and flanking intronic sequences of *MSX1*

| **Exon** | **Forward (5’-3’)** | **Reverse (5’-3’)** |
| --- | --- | --- |
| **1** | CCTTATTAGCAAGTTCTCTGGGGA | AGGACAGGGTGCCCAGACATC |
| **2** | TACTACTTCTTGGGCTGATCATGC | CAGTGTGAGGGTTAAAGGGAAGG |

Table S3. Primers amplifying coding exons and flanking intronic sequences of *AXIN2*

| **Exon** | **Forward (5’-3’)** | **Reverse (5’-3’)** |
| --- | --- | --- |
| **2** | CCTGCTGACTTGAGAGAGACAGAG | CCCATCCACCATACTTAAAACATCTGC |
| **3** | TGGGATAGTGTGCTGGGTTGTC | CAGCTGAGGATGACAGACGATTCT |
| **4** | TATGACAGTAGCCCATGTGATGTG | CCCATTCCACCACCCATTTCTTTT |
| **5** | CATCCACTCTACACCCGAACATG | CACATGCGCACACCCTAACG |
| **6** | GTAGGGAGCCGAATGTTGCAAC | CTGTAATGCGGCTCCCACCTC |
| **7** | CCCCGACTTGCTGAATTGTCTGAT | AGTCACATTTGTATTCCGCGGACT |
| **8** | GCACTGACCCCTGTTCATGTTATG | GTTTGAGACCCAGGCAGAAAGAGA |
| **9** | CGTAAGGATGGAAGTCGGGTTC | GGCAACATCTACGTTTACTGTTCCT |
| **10** | CAATTGCAGCCCTAGTGTTTGGAT | TGGTTCTGAGCAAACAAACTGAGA |
| **11** | CAGTCCCAGCTGCCGTCTTAAA | CTCACAGCCAAGACAGTTCACAAG |

Table S4. Primers amplifying coding exons and flanking intronic sequences of *LRP6*

| **Exon** | **Forward (5’-3’)** | **Reverse (5’-3’)** |
| --- | --- | --- |
| **1** | CCTTTCTTTCTTCTCTCGCTGGGA | CCCCGAACCCCACCAACTTTC |
| **2** | TGTGTGTGAAGTTTAGATGGTCTCCTA | GGGTGGTGTATGTCAGTGGAGAAA |
| **3** | CAGAGTATTTGATTGATATCTGCTCT | GACTATTCTTCTTCCCCTCTGG |
| **4** | TGGGAGAGGTGACGTTATGATAGAAG | CCTGATCCTCACTAAAGCCAGACA |
| **5** | TGCATGACATTTGGTAATAATTTTGGCT | CCCAAAGCAGTATAACCTAGAGAGCT |
| **6** | TCTAAAGAGAGTGCACATCCTTTTGT | ACAACACCCAAAGTCAGCAAAC |
| **7** | ACCAGTGTAATAGGAGGGATGGAT | GATCAGCAGCCATTTCTCATACCA |
| **8** | GGGAAAAGTGGTCAAATAGAGGCTTA | AATTTACACTGCTGACTATCTCCATCT |
| **9** | GGGAGCAAGACATAATCATAGGCAT | CAAACAATGAGGGAGGTGGGT |
| **10** | ATCCTCTTGCCCCTGACAAA | ACTATGCCATGTTCCCCGAA |
| **11** | TGTAGCCGTGATTTTGTTTAACCCA | AGCCACTGATATTTGCATGGAAAGA |
| **12** | TTGCCAGCAAGACACTTGTATT | GCTGCTTTCTCTCATTCTAGCT |
| **13** | TTGGATGATAGAGGATGTAGGGTAACTA | GAAACAGAGTGGTTGGTGAGTCC |
| **14** | ACTCCTTAAGACTTGAGAGAGCCT | TGGAGCACAGGACACTTAAAGG |
| **15** | GAGCCACTGTGCCTGGTCAAAA | CCAACCAACCTGATGCTGACTACA |
| **16** | TAGAATATTTACTGCACATTGGGC | GAAAGTCTTCAAGGAAACAGAGTT |
| **17** | AAAGAGTCCAAGCTGATTATACATTT | TGGAACACACGCAACCAATTAAGT |
| **18** | ACTTCTGTGTGACCATGATTGTGT | ACTGAAGTTTAAACAACTGAATGGGAAA |
| **19** | GCACCTTTTGATTCTTGCCAGAG | AGGAAATCTCGATAAGTAAACCTCACA |
| **20** | TCCTATTAGCAAGCCCTCTTTTGAT | TGGTTTCAGACAGACTCTAGGTAGTATT |
| **21** | GGGAGCTATTCTTGGCCTTGTTCT | GGATGGTGGTGTGTGGTAAGTCC |
| **22** | TCTTAGGGAATGAGGAAGCCATAG | TACTCATTTGGGGCTATATCAGGT |
| **23** | GCGAATCTGCCATTGAAAATTGCC | CCCCTCCAGATCTCAACCAAATTT |

Table S5. Primers amplifying coding exons and flanking intronic sequences of *WNT10B*

| **Exon** | **Forward (5’-3’)** | **Reverse (5’-3’)** |
| --- | --- | --- |
| **2-3** | CTGAACCCGCATCAAGTCTCCC | AAACCATCCCTTCCCGCCTC |
| **4** | TTCTTTCTGCCTCCACACTCTCAG | AACTCTAACCAGGCCTCAAAAGCT |
| **5** | ATTTGTCCCTCCCTGTGTTCTCTG | TTTAAGCTTCCAGGGACCAAGAGT |
