# Supplementary figures and images for: Novel EDA or EDAR Mutations Identified in Patients with X-Linked Hypohidrotic Ectodermal Dysplasia or Non-Syndromic Tooth Agenesis
Source: Genes (Basel). 2017 Oct 5;8(10):259. doi: 10.3390/genes8100259 (PMC5664109; doi:10.3390/genes8100259)

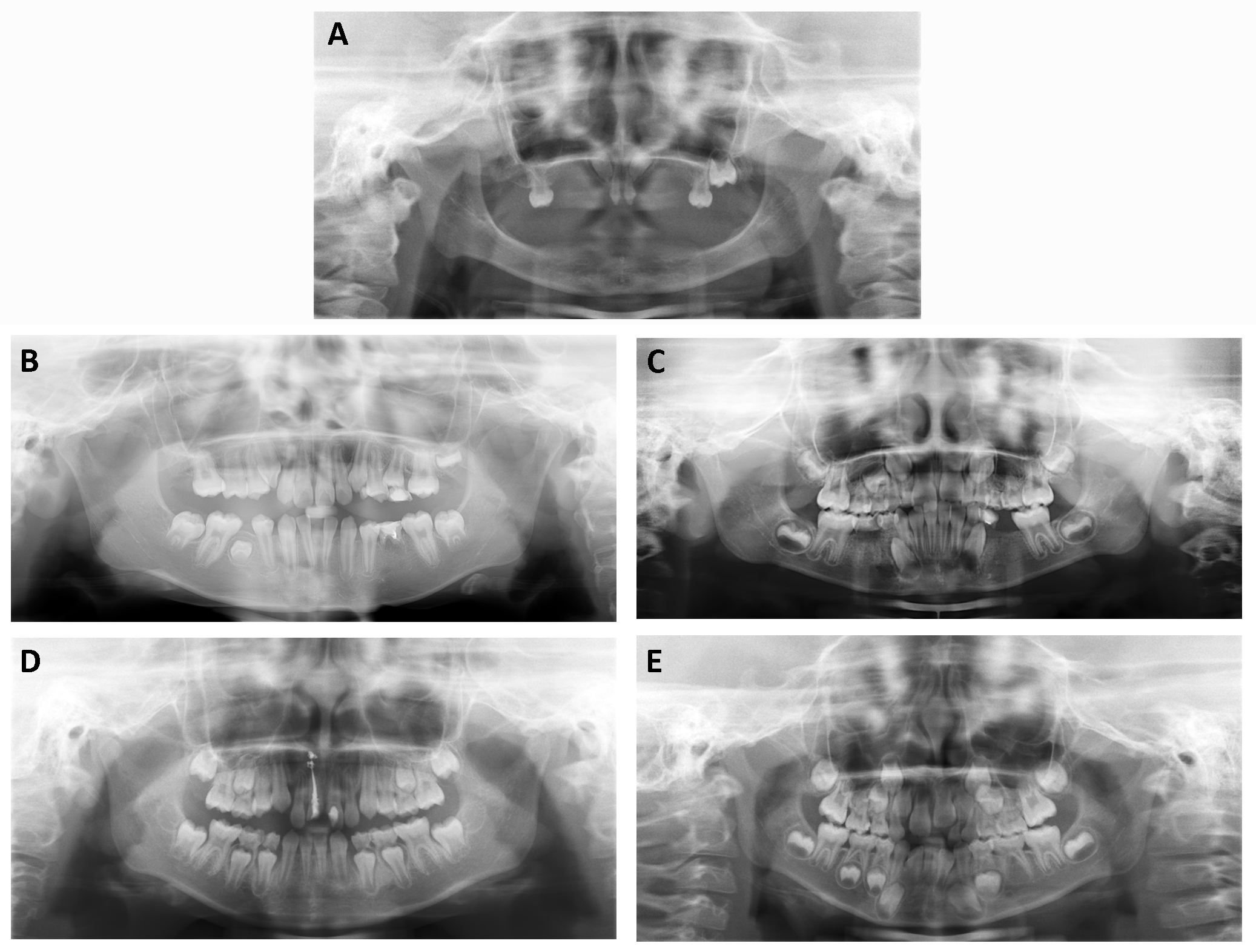

Supplement: Supplementary file 1 [file genes-08-00259-s001.zip › Supplementary Materials/Fig 1.tif]
